# Endotoxin in drainage fluid as an early and predictive marker of anastomotic leakage after colorectal surgery

**DOI:** 10.1007/s00595-025-03106-x

**Published:** 2025-07-23

**Authors:** Takashi Matsunaga, Toru Miyake, Takeru Maekawa, Fumie Tsukaguchi, Toru Obata, Tomoharu Shimizu, Masaji Tani

**Affiliations:** 1 Department of Surgery, Shiga University of Medical Science, Seta, Tsukinowa-cho, Otsu, Shiga 520-2192 Japan; 2 Department of Clinical Laboratory, Shiga University of Medical Science Hospital, Seta, Tsukinowa-cho, Otsu, Shiga 520-2192 Japan; 3 Medical Safety Section, Shiga University of Medical Science Hospital, Seta, Tsukinowa-cho, Otsu, Shiga 520-2192 Japan

**Keywords:** Endotoxin, Anastomotic leakage, Colorectal surgery

## Abstract

**Purpose:**

To assess the predictive value of the endotoxin (ET) assay for the detection of anastomotic leakage (AL) after colorectal surgery (CRS).

**Methods:**

ET levels in the drainage fluid were measured using endotoxin scattering photometry (ET-ESP) and turbidimetric (ET-TUB) assays on postoperative day (POD) zero, POD1 and POD3, comparing tumor necrosis factor (TNF)-α.

**Results:**

AL was observed in 8 (4.9%) of the 162 patients. ET-ESP, ET-TUB, and TNF-α levels on POD0 and serum C-reactive protein (CRP) on POD1 were significantly elevated in the AL group. The area under the receiver operating characteristic curve (AUROC) for ET-ESP level (0.903) on POD0 showed early and better predictive performance for AL compared to that for ET-TUB (0.869, *p* = 0.230) and TNF-α (0.758, *p* = 0.034) levels on POD0; the AUROC for CRP level (0.711) on POD1 was inferior to other parameters. In subgroup analysis, five (3.7%) of 136 patients with colorectal cancer (CRC) developed AL. Additionally, the ET-ESP level on POD0 showed relatively good predictive performance for AL after CRC (AUROC: ET-ESP [0.871], ET-TUB [0.840], and TNF-α [0.737] on POD0).

**Conclusion:**

ET levels in drainage fluid, especially those measured using ESP, on POD0 may have an early predictive ability to detect AL post-CRS.

## Introduction

Anastomotic leakage (AL) after colorectal surgery (CRS) is a life-threatening postoperative complication that leads to intraabdominal sepsis secondary to peritonitis. Despite the development of critical care treatment strategies for intraabdominal sepsis, the reported mortality rate remains 2–16.4% [1]. In total, 39% of patients who underwent surgery for AL post-CRS were reported to have a stoma at the 1-year follow-up, resulting in restrictions in daily life activities or decreased quality of life [2]. Moreover, AL after curative colorectal cancer (CRC) surgery is correlated with increased local recurrence and reduced long-term patient survival [3]. The safety of colorectal surgical procedures is certainly improving; however, recent reports have also shown a relatively high incidence of AL after CRS for CRC and inflammatory bowel disease (IBD), ranging from 2.8 to 30% [1, 4–8]. The prediction and early diagnosis of AL are important to improve patient prognosis and quality of life. The standard diagnostic modalities for AL involve clinical symptom monitoring and imaging examinations such as CT; therefore, AL is usually diagnosed 6–12.7 days postoperatively [6, 9–13]. However, early and useful predictive markers for AL detection in clinical settings are unavailable.

The pathophysiology of AL involves leakage of luminal contents from surgical anastomosis of the intestinal tract into the intraabdominal cavity. The intestinal lumen contains abundant Gram-negative bacteria. Endotoxins (ET) are a major component of the cell walls of these bacteria. In our previous study, we observed that plasma ET levels were predominantly elevated in patients with septic shock due to perforated peritonitis [14]. Junger et al. previously showed that ET levels were significantly higher in the drainage fluid of patients with AL relative to those without AL. They concluded that ET-related parameters allowed reliable detection of AL on postoperative day (POD) 3 after surgery [15]. In their study, ET was measured using ET-nonspecific limulus amebocyte lysate (LAL) reagents, which react with ET and β-glucan. ET-specific LAL reagents have been developed [16]. To the best of our knowledge, no studies have reported ET levels in drainage fluid using ET-specific LAL reagents as predictive markers for AL after CRS.

Recently, Obata et al. developed a novel LAL assay using a laser light-scattering particle-counting method, termed endotoxin scattering photometry (ESP). The standard turbidimetric assay measures the turbidity of gel clots formed when the LAL reagent reacts with endotoxins. The ESP assay measures the formation of coagulin particles, which are the precursors of gel clots, under continuous agitation. Therefore, the ESP assay can theoretically detect ET more rapidly than the turbidimetric assay [17]. The clinical potential of the ESP assay has been reported in patients with sepsis and septic shock undergoing emergency gastrointestinal surgery [14, 18].

We hypothesized that ESP assays using ET-specific LAL reagents have the potential to predict AL after CRS at an earlier time point than previously reported. To clarify this hypothesis, we measured ET levels in the drainage fluid of the abdominal cavity using ESP and turbidimetric assays with ET-specific LAL reagents, comparing tumor necrosis factor (TNF)-α as an inflammatory cytokine.

## Methods

### Patients

This prospective, observational, clinical study was conducted at the Department of Surgery, Shiga University of Medical Science. We included 215 consecutive patients who underwent elective CRS with single anastomosis and drain placement between June 2021 and February 2024. In total, 53 patients were excluded from the study (inadequate sample collection, *n* = 15 and other reasons [dementia and other mental diseases, *n* = 4; refusal to participate in the research, *n* = 15; lack of consent *n* = 19). Finally, 162 patients were included in the study. Written informed consent was obtained from all patients prior to surgery. The study protocol was approved by the Ethics Committee of the Shiga University of Medical Science (No. R2020-202).

### Preoperative preparation and surgical procedure

Preoperatively, all patients underwent mechanical bowel preparation with laxatives a day before surgery and received metronidazole 500 mg twice daily as an oral antibiotic. All patients were treated according to the Japanese Society for Cancer of the Colon and Rectum (JSCCR) guidelines 2019, for the treatment of CRC [19], JSCCR guidelines 2022 for the treatment of CRC, and Evidence-based Clinical Practice Guidelines for Inflammatory Bowel disease 2020 [20]. Functional end-to-end (FEEA) and delta anastomoses were performed for colectomies using a linear stapler, the double-stapling technique (DST) for sigmoidectomy, Hartmann’s reversal, and rectal resections using linear and circular staplers. Coloanal anastomosis following intersphincteric resection for very low rectal cancer and ileal pouch-anal anastomosis for total proctocolectomy or completion proctectomy were performed using the hand-sewn technique. After anastomosis, an intraabdominal lavage with approximately 1 L of saline solution was poured into the abdominal cavity through a small incision, and an 8-mm-diameter surgical drain was placed in the peritoneal cavity near the anastomosis. The diverting stoma was created at the surgeon’s discretion.

### Perioperative follow-up and definition of anastomotic leakage

The patients were monitored for clinical signs of AL and routine laboratory examinations were conducted. In cases of suspected AL, further investigations, including computed tomography (CT) or radiographic contrast studies, were performed to confirm the presence of AL. Patients received appropriate treatment according to AL grade. AL is defined as communication between the intraabdominal and extraluminal compartments owing to a defect in the integrity of the intestinal wall at the site of anastomosis. AL was diagnosed based on the following criteria: (1) apparent leakage of gas/pus/feces from the abdominal or pelvic drain; (2) anastomotic defect in the colon and rectum confirmed via CT with contrast media; and (3) AL confirmed during reoperation [21].

### Sample collection

Drainage fluid was collected into a drainage bottle that was closely connected to the abdominal drain and discarded every 8 h (7:00, 15:00, 23:00) after surgery by trained medical staff. The properties and amount of the drainage fluid were monitored. A 2 ml sample of drainage fluid was collected from the drainage bottle into pyrogen-free polyester tubes (FUJIFILM Wako Pure Chemical Corporation, Osaka, Japan) immediately after surgery (approximately 1–5 h after surgery, postoperative day [POD] zero), at 15:00 on POD1 and POD3. The supernatant of the drainage fluid was obtained by centrifugation (150 × *g*, 10 min, 4 °C) and stored at −80℃ until analysis, as previously described [12].

All glassware items were sterilized by dry heating at 250 °C for at least 3 h to make them endotoxin-free. Sterile ET-free plastic pipette tips and pyrogen-free polyester tubes (FUJIFILM Wako Pure Chemical Corporation) were used to prepare samples. ET-specific LAL reagents, Endotoxin-Single Test Wako, and sample pretreatment solution (FUJIFILM Wako Pure Chemical Corporation) were used to avoid the influence of β-D-glucan on the ET-induced coagulation pathway [22].

### ESP assay, turbidimetric assay and TNF-α assay

The ESP assays were performed using a third-generation backscatter measurement system. The supernatant of the sample was diluted 1:100 with an ESP diluted sample pretreatment solution as previously described [17]. The mixture was heated to 70 °C for 10 min and then cooled on ice until the assay. A 0.63 mL aliquot of the pretreated sample was then mixed with the LAL reagent. Each reaction glass tube was maintained at 37 °C under continuous agitation using a mini-spin bar. The intensity of backscattered light was measured to detect the formation of coagulin particles. An original computer software program was used to display the time course of the alteration in the intensity of the total backscattered light as two-dimensional data. Because the reaction time until the appearance of coagulin particles is proportional to the ET concentration, the ET concentration was calculated in relation to the calibration curve of individual lots of LAL reagents. The third-generation backscatter measurement system was adjusted to enable detection of standard latex particles as small as 20 μm. The detection limit of the ESP assay was 0.1 pg/mL at 70 min.

The ET concentration of the turbidimetric assay in the drainage fluid was also measured turbidimetrically using the same LAL reagent according to the manufacturer’s instructions [14]. For sample preparation, 0.1 mL the supernatant sample and 0.9 mL and sample pretreatment solution were mixed. The mixture was heated to 70 °C for 10 min and then cooled on ice until the assay. The LAL reagent and 0.2 mL of the pretreated sample was mixed in a test tube and incubated at 37 °C under settled conditions to measure ET in a tube reader LIMUSAVE MT-7500 (FUJIFILM Wako Pure Chemical Corporation). The gelation time was defined as the time required for the transmittance ratio of the reaction mixture to decrease by 92%. The ET level was assessed from the gelation time of the sample in relation to the calibration curve of individual lots of the LAL reagents. The detection limit of the turbidimetric assay was 2.1 pg/mL at 90 min.

TNF-α levels in the drainage fluid were measured using an enzyme-linked immunosorbent assay (ELISA), according to the manufacturer’s instructions (Human TNF-α ELISA Kit, FUJIFILM Wako Pure Chemical Corporation).

### Statistical analysis

Statistical analyses were performed using IBM SPSS Statistics for Macs (ver. 29, IBM Corp., Armonk, N.Y., USA). Comparison of receiver operating characteristic curves (ROCs) among the parameters was performed using EZR (Saitama Medical Center, Jichi Medical University, Saitama, Japan), which is a graphical user interface for R (ver. 4.2.0; The Foundation for Statistical Computing, Vienna, Austria) [23]. Categorical variables were analyzed using Fisher’s exact test and the chi-square test, and comparisons of the three groups were adjusted using the Bonferroni method. Numerical variables were evaluated using the Mann–Whitney U and Wilcoxon signed-rank sum tests. Data are expressed as the median and interquartile range, unless otherwise noted. ROCs were constructed to identify the best cutoff points for various parameters to predict AL after CRS. Statistical significance was set at *p* < 0.05 (two-tailed). The sample size was determined using a G*Power analysis [24]. The statistical power of this study was calculated to include 178 individuals in both groups (AL group = 14, non-AL group = 162) with a confidence interval of 95%, two-tailed, normal parent distribution, effect size (d) of 0.8, α error of 5%, and 1-β error of 80%, assuming an 8% incidence of AL. Therefore, we planned to recruit at least 210 patients, accounting for a dropout rate of 20% during the study period.

## Results

AL was observed in 8 (4.9%) of 162 patients after CRS. Patients in the AL group were younger than those in the non-AL group. The AL group included more patients with IBD than the non-AL group. Differences in the surgical and anastomotic procedures were observed between the AL and non-AL groups. The operative time was longer in the AL group than in the non-AL group (Table 1).

**Table 1:** Patient characteristics

	AL group (*n* = 8)	non-AL group (*n = *154)	*p* value
Sex (Male/Female)	7 (88%)/1 (12%)	85 (55%)/69 (45%)	0.139
Age	60 (52–67)	71 (62–76)	0.022
BMI (kg/m^2^)	22.6 (20.4–25.4)	22.3 (19.9–24.8)	0.754
ASA-PS (1/2/3)	2 (25%)/5 (63%)/1 (12%)	15 (10%)/113 (73%)/26 (17%)	0.387
Serum albumin (g/dL)	4.0 (3.8–4.2)	3.9 (3.6–4.2)	0.436
PNI	47.0 (44.8–49.4)	46.4 (42.3–50.3)	0.720
HbA1c (%)	5.7 (5.3–7.1)	5.8 (5.5–6.3)	0.820
Smoking (never/former/current)	2 (24%)/3 (38%)/3 (38%)	74 (48%)/61 (40%)/19 (12%)	0.110
Disease (Cancer/IBD/Others)	5 (62%)/3 (38%)*/0 (0%)	131 (85%)/13 (8%)/10 (7%)	0.024
Operation (colectomy/proctectomy/TPC or CPC)	1 (12%)*/4 (25%)/3 (38%)*	80 (52%)/62 (40%)/12 (8%)	0.007
Combined resection of adjacent organs	0 (0%)	6 (4%)	1.000
Approach (Open/Lap/Robotic)	0 (0%)/6 (75%)/2 (25%)	12 (8%)/106 (69%)/36 (23%)	0.714
Anastomosis (Handsewn/Stapled)	3 (38%)/5 (62%)	14 (9%)/140 (91%)	0.039
Diverting stoma	3 (38%)	36 (23%)	0.400
Operating time (min)	374 (343–455)	287 (229–380)	0.049
Blood loss (mL)	0 (0–178)	2 (0–100)	0.937
Transfusion	0 (0%)	14 (9%)	1.000
Preoperative preparation (MBP only/MBP + OA/none)	0 (0%)/8 (100%)/0 (0%)	15 (10%)/137 (89%)/2 (1%)	0.611

Continuous parameters are expressed as the median (25th–75th percentile). Categorized data are expressed as number (percent). *AL* anastomotic leakage, *BMI* body mass index, *ASA-PS* American society of Anesthesiologists physical status, *PNI* prognostic nutritional index, PNI was calculated as 10 × (serum albumin levels) + 0.005 × (total lymphocyte count)

*HbA1c* hemoglobinA1c, *IBD* inflammatory bowel disease, *TPC* total proctocolectomy, *CPC* completion proctectomy, *Lap* laparoscopic, *MBP* mechanical bowel preparation

*P < 0.05 vs. non-AL group by the Bonferroni method

**Fig. 1:**
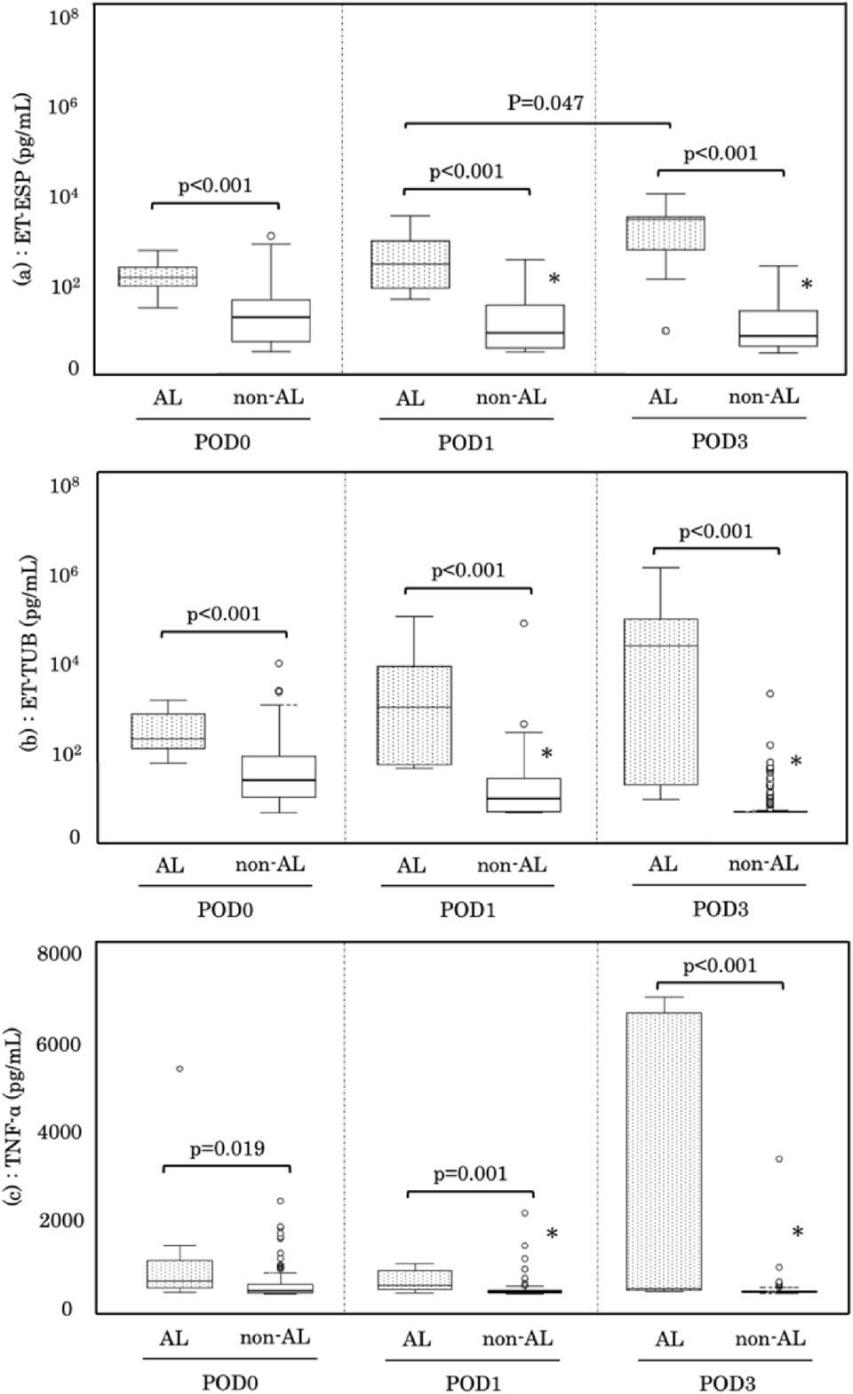
Alteration in ET-ESP (**a**), ET-TUB (**b**) and TNF-α (**c**) levels in drainage fluid between the AL and non-AL groups. ET-ESP, ET-TUB, and TNF-α levels in patients with AL were significantly elevated in comparison to those in patients without non-AL on POD0, POD1 and POD3. ET-ESP, ET-TUB, and TNF-α levels in patients without non-AL on POD1 and POD3 were significantly lower than those in patients without non-AL on POD0. Data are shown as the median (25th–75th percentile). Dotted boxplots, AL group; white boxplots, non-AL group. *p < 0.05 vs. on POD0 in the non-AL group. *ET* endotoxin, *ESP* endotoxin scattering photometry, *TUB* turbidimetric assay, *AL* anastomotic leakage, *POD* postoperative day

**Fig. 2:**
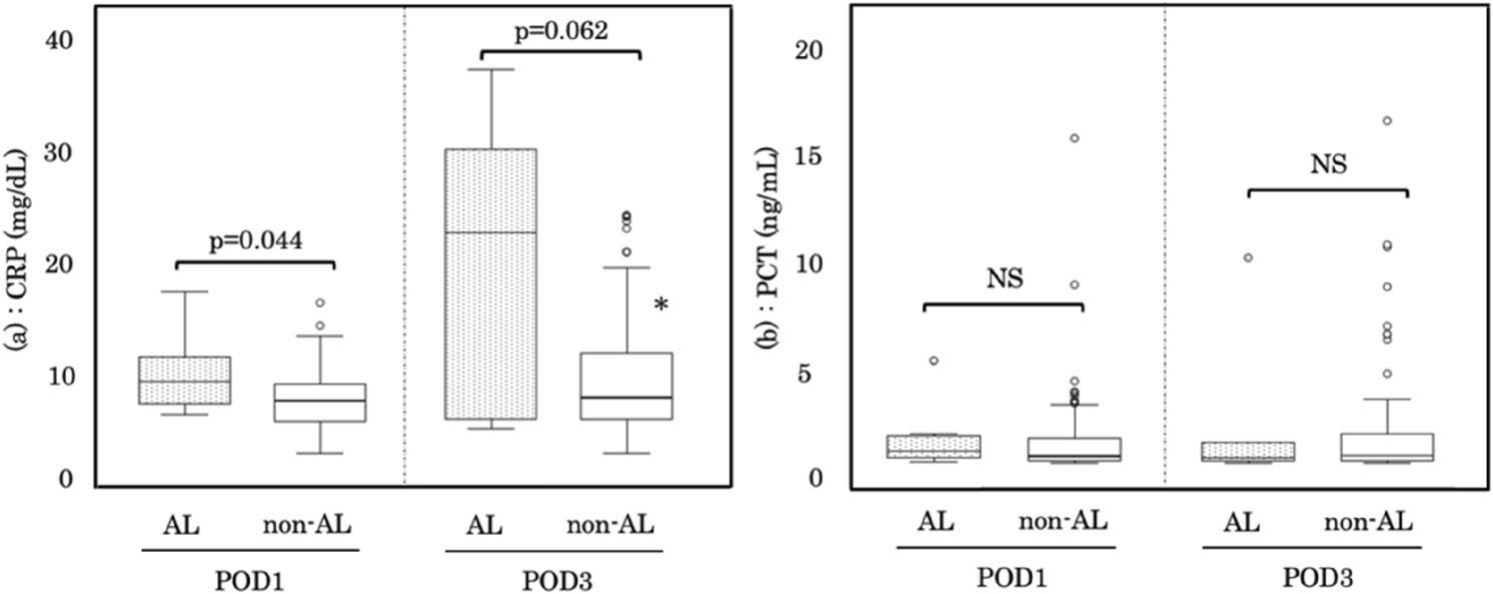
Alteration in serum CRP (**a**) and PCT (b) levels between the AL and non-AL groups. Serum CRP levels in patients with AL on POD1 were significantly higher than those in patients without AL. Serum CRP levels in the non-AL group on POD1 were significantly increased in comparison to those in in the non-AL group on POD0. No significant difference was observed in PCT levels between the AL and non-AL groups on POD1 and POD3. Data are shown as the median (25–75th percentile). Dotted boxplots, AL group; white boxplots, non-AL group. *p < 0.05 vs. non-AL group on POD 1. *CRP* C-reactive protein;, *PCT* procalcitonin;, *AL* anastomotic leakage;, *POD* postoperative day

The ET levels measured using the ESP assay (ET-ESP) on POD0 were significantly higher in the AL group than in the non-AL group (median 184.3 pg/mL vs. 9.4 pg/mL, *p* < 0.001). ET-ESP in the AL group was significantly increased on POD3 than that on POD1 (Fig. 1a). Significant elevations in ET levels measured using the turbidimetric assay (ET-TUB) on POD0 were observed in the AL group in comparison to those in the non-AL group (median 495.8 pg/mL vs. 21.1 pg/mL, *p* < 0.001; Fig. 1b). TNF-α levels in the drainage fluid on POD0 were also significantly higher in the AL group than in the non-AL group (median 315.8 pg/mL vs. 81.7 pg/mL, *p* = 0.019; Fig. 1c). The ET-ESP, ET-TUB, and TNF-α levels in the non-AL group significantly decreased in a time-dependent manner (Fig. 1 a, b, c). In postoperative blood examination, serum C-reactive protein (CRP) levels on POD1 were significantly higher in the AL group than in the non-AL group (median 8.1 mg/dL vs. 6.1 mg/dL, *p* = 0.044; Fig. 2a). However, plasma procalcitonin (PCT) levels were not significantly different between the AL and non-AL groups (Fig. 2b). A subgroup analysis for the comparison of ET-ESP levels between patients with CRC and IBD is shown in Table 4. Changes in ET-ESP levels after surgery in the AL group were comparable between patients with CRC and those with IBD (Table 4).

**Fig. 3:**
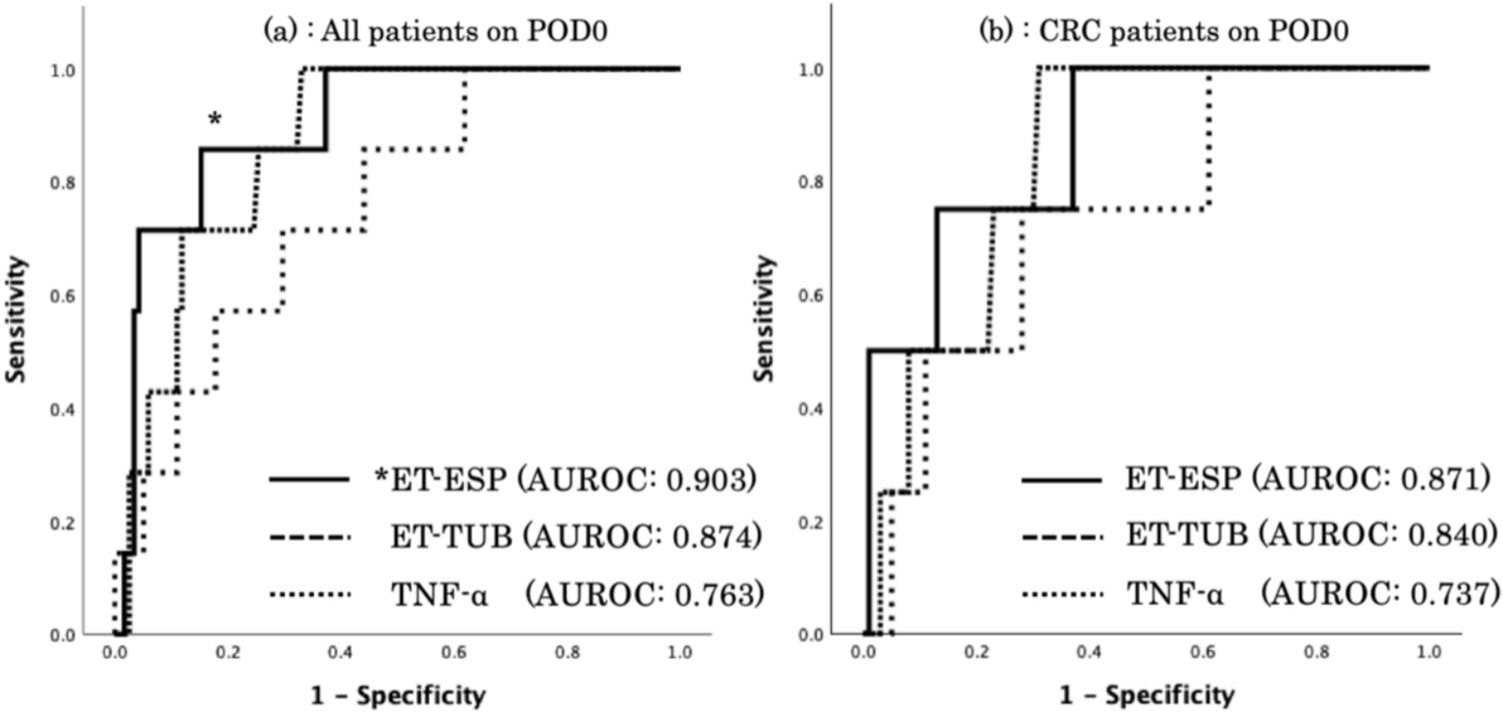
Comparison of ROCs for ET-ESP, ET-TUB and TNF-α levels on POD0 in all patients (**a**) and patients with CRC (**b**). The ET-ESP level on POD0 was an early predictor and showed the best predictive performance relative to ET-TUB and TNF-α in all patients and in those with CRC. *p < 0.05 ET-ESP vs. TNF-α on POD0 in all patients. *ROC* receiver operating characteristic, *ET* endotoxin, *ESP* endotoxin scattering photometry, *TUB* turbidimetric assay, *POD* postoperative day, *CRC* colorectal cancer, *TNF-α* tumor necrosis factor-α

The early and predictive performance of these parameters in drainage fluids on POD0 to detect AL after CRS is shown in Fig. 3. The ET-ESP level on POD0 showed the best predictive performance; however, no significant difference was observed between the area under the receiver operating characteristic curve (AUROC) of ET-ESP and ET-TUB levels on POD0 (AUROC: 0.903 vs. 0.874, *p* = 0.230). Furthermore, ET-ESP was superior to TNF-α for predicting AL on POD0 (AUROC: 0.903 vs. 0.763, *p* = 0.034). No significant difference was observed between the ET-TUB and TNF-α levels on POD0 (AUROC: 0.874 vs. 0.763, *p* = 0.110; Fig. 3a). In the subgroup analysis of patients with CRC, 5 (3.7%) of 136 patients had AL after surgery for CRC (Table 4). The ET-ESP level on POD0 also exhibited good predictive performance for AL after surgery for CRC, and no significant differences in AUROC were observed among ET-ESP, ET-TUB, and TNF-α levels on POD0 (AUROC: 0.871 vs. 0.840 vs. 0.737; Fig. 3b).

Details of the predictive performance of these parameters in the drainage fluid for AL are shown in Table 2. Additionally, the ET-ESP and ET-TUB levels on POD1 had excellent predictive values (AUROC: 0.950 vs. 0.940, *p* = 0.221). The AUROCs for ET-ESP, ET-TUB, and TNF-α levels on POD1 and POD3 increased in comparison to those on POD0. Although the plasma CRP levels on POD1 and POD3 showed relatively good predictive performance, they were inferior to those of ET-ESP and ET-TUB levels in the drainage fluid on POD1 and POD3 (Table 2).

**Table 2:** AUROC, sensitivity and specificity among various parameters for prediction of AL

Samples	Sampling date	Assay	AUROC	95% CI	Optimal cutoff value	Sensitivity	Specificity
Drainage fluid	POD0	ET-ESP	0.903	0.808–0.999	73.9 pg/mL	85.7%	85.1%
		ET-TUB	0.874	0.787–0.961	423.3 pg/mL	71.4%	88.3%
		TNF-α	0.763	0.602–0.924	182.3 pg/mL	71.4%	71.7%
	POD1	ET-ESP	0.950	0.897–1.003	84.5 pg/mL	87.5%	91.3%
		ET-TUB	0.940	0.885–0.996	50.4 pg/mL	100%	82.8%
		TNF-α	0.842	0.696–0.988	134.5 pg/mL	75.0%	86.7%
	POD3	ET-ESP	0.930	0.808–1.051	145.0 pg/mL	85.7%	96.9%
		ET-TUB	0.948	0.892–1.004	5.0 pg/mL	100%	82.0%
		TNF-α	0.852	0.725–0.978	119.4 pg/mL	75.0%	87.9%
Blood	POD1	CRP	0.711	0.543–0.880	7.4 mg/dL	62.5%	70.8%
		PCT	0.616	0.455–0.778	0.5 ng/mL	75.0%	51.3%
	POD3	CRP	0.726	0.439–0.989	15.8 mg/dL	66.7%	89.9%
		PCT	0.485	0.214–0.757	0.9 ng/mL	40.0%	69.1%

*AUROC* area under receiver operating characteristic curve, *CI* confidence interval, *POD* postoperative day, *ET* endotoxin, *ESP* endotoxin scattering photometry, *TUB* turbidimetric, *TNF-α* Tumor Necrosis Factor-α, *CRP* C-reactive protein, *PCT* procalcitonin

The median time of AL onset in the 8 patients was POD4 (min. POD2 – max. POD7). Two of the 8 patients with ISGRC Grade C required reoperation, and 6 showed improvement with conservative treatment, including antibiotics and percutaneous drainage. None of the patients required treatment in the intensive care unit or died within 30 days of surgery. Bacterial cultures were obtained from ascites fluid in 5 of the 8 patients, and Gram-negative bacteria were identified in all samples. The maximal point of Sequential Organ Failure Assessment (SOFA) score was 5, even in patients with ISGRC Grade C AL who underwent relaparotomy (Table 3).

**Table 3:** Details of the microbial characteristics and clinical course of cases with anastomotic leakage

Disease	Date of onset	Site	Microorganism	ISGRC grade	SOFA score (maximal points)	Postoperative hospital stay (days)
Rectal cancer	POD7	ascites	*Escherichia coli 2* + *Clostridiumm perfringens 3* + *Bacteroides thetaiotaomicron 3* + *Enterococcus species 3* + *Bifidobacterium species 3* +	B	0	40
UC	POD4	ascites	*Enterobacter aerogenes 2* + *Enterococcus gallinarum* < +	B	1	38
UC	POD5	wound	*Corynebacterium striatum 2* +	A	0	9
Rectal cancer	POD3	ascites	*Morganella morganii 3* + *Enterobacter species 3* + *Enterococcus faecalis 3* + *Enterococcus avium 3* +	B	1	25
Rectal cancer	POD2	ascites	*Enterobacter aerogenes 2* + *Esherichia coli* < + *Enterococcus faecalis* < + *Candida glabrata* < +	C	5	22
Rectal cancer	POD4	blood	negative	A	1	27
UC	POD4	ascites	*Enterobacter cloacae* < + *Enterococcus faecalis* < +	A	1	21
Rectal cancer	POD4	blood	*Enterococcus faecalis* < +	C	2	36

Patients with rectal cancer received double stapling technique for reconstruction without diverting stoma. Patients with UC received ileal pouch-anal anastomosis for reconstruction with diverting stoma

*ISGRC* international study group of rectal cancer, *SOFA* sequential organ failure assessment, *UC* ulcerative colitis, *POD* postoperative day

## Discussion

To the best of our knowledge, this is the first study to investigate the predictive value of ET-ESP and ET-TUB in drainage fluids in the detection of AL after CRS. To our knowledge, no study has reported a significant elevation in ET levels in the drainage fluid immediately after surgery as a predictor of AL after CRS. The early prediction of AL on POD0 may help guide timely interventions to prevent deterioration of the patient’s general condition after the clinical onset. This could include enhanced monitoring, delaying initiation of oral intake, extending antibiotic administration, or modifying it from prophylactic to therapeutic. Moreover, postponing the removal of intra-abdominal or trans-anal drains may reduce the incidence of extraperitoneal AL and the rate of reintervention after anterior rectal resection. Nevertheless, vacuum-assisted wound therapy has been successfully adapted as an endoscopic approach for managing AL after upper and lower gastrointestinal surgeries, known as endoscopic vacuum therapy (EVT). A recent systematic review and meta-analysis showed that EVT is a potentially feasible treatment option with manageable risks for selected patients with AL after colorectal surgery [25]. If EVT is initiated based on early detection of elevated ET levels in drainage fluid on POD0, it may help prevent further disruption of anastomotic healing before the clinical manifestation of AL (Table 4).

**Table 4:** Comparison of the ET-ESP levels between CRC and IBD patients

Sampling date	ET-ESP in CRC patients (pg/mL)		*p* value	ET-ESP in IBD patients (pg/mL)		*p* value
	AL group (*n* = 5)	non-AL group (*n* = 131)		AL group (*n* = 3)	non-AL group (*n* = 13)	
POD 0	171.3 (61.3–327.7)	9.4 (2.0–31.7)	0.012	184.3 (162.0–692.1)	6.0 (3.3–414.6)	0.243
POD 1	86.2 (82.7–780.6)	3.3 (1.3–22.6)	0.001	937.3 (599.4–6993.3)	47.3 (7.6–122.7)	0.025
POD 3	5329.2 (124.3–10,501.2)	3.0 (1.6–13.0)	0.013	14,474.9 (12,226.7–38,179.7)	51.7 (1.6–57.8)	0.017

Continuous parameters are expressed as the median (25–75th percentile)

*AL* anastomotic leakage, *ET* endotoxin, *ESP* endotoxin scattering photometry, *UC* ulcerative colitis, *POD* postoperative day

According to our findings, increased ET levels around the anastomotic site in the early postoperative phase may be related to AL development; however, the mechanism of this early elevation remains uncertain. ET levels in the drainage fluid on POD0 and POD1 were at the “pg/mL” range. This elevation may be due to contamination of the intestinal content during surgical procedures or possible microscopic leakage derived from the small communication between intra- and extra-luminal bowel compartments after anastomosis. Sparreboom et al. categorized the etiology of AL into three major components: communication, infection, and healing disturbances [26]. They proposed that communication due to an anastomotic defect causes leakage of colonic contents into the abdominal or pelvic cavity, indicative of bacterial infection at the anastomotic site, which may further worsen healing at the site of anastomosis. Bacterial ET present in the abdominal cavity can activate inflammatory responses in inflammatory cells or intestinal epithelial cells, which produce inducible nitric oxide synthase (iNOS) [27] and matrix metalloproteinase (MMP)−9 [28–30]. The decrease in collagen deposition by iNOS [31] and degradation of extracellular collagen by MMP-9 [32] negatively affect anastomotic healing and cause subsequent enlargement of the communication of bowel compartments. Additionally, ET levels in the drainage fluid on POD3 increased to the “ng/mL” range, indicating further enlargement of bowel communication and the presence of macroscopic AL. The timing of “ng/mL” ET elevation corresponds to the median POD4 onset of AL observed in our study. Thus, elevated ET levels in the drainage fluid may either directly contribute to AL development or notably impair the healing process.

We did not identify any statistically significant differences in predictive performance between ET-ESP and ET-TUB. ET levels in the drainage fluid were higher than those in the plasma of patients with sepsis, as shown in our previous study [14]. Given that the logarithmic values of the reaction time and ET level in the LAL reagent have a linear proportional relationship, the superiority of ESP sensitivity decreases in the high concentration range above a few pg/mL. Junger et al. demonstrated that ET levels measured using ET-nonspecific LAL reagents in the drainage fluid significantly increased on POD1; however, ET levels in the non-AL group were also high, suggesting the influence of false-positive reactions of ET-nonspecific LAL reagents owing to β-glucan. Kimura et al. demonstrated an increase in plasma and peritoneal levels of β-glucan in the early postoperative period when measured using ET-nonspecific reagents, likely due to contamination from surgical materials, such as gauze [33]. In this study, we measured ET levels in the drainage fluid of the abdominal cavity using ET-specific LAL reagents. Moreover, it is reasonable to speculate that delta anastomosis and FEEA may facilitate the release of intestinal lumen contents, potentially leading to higher ET levels in comparison to DST; however, ET-ESP, ET-TUB, and TNF-α levels in patients with DST showed no significant differences in comparison to those in patients with delta anastomosis and FEEA (data not shown).

Previous studies have explored various biomarkers for the early detection of AL, such as markers of ischemia, inflammation, and microbiological biomarkers in drainage fluid. Most studies reported that the detection time points for these markers were POD1–5 [34]. From the perspective of early prediction, Pasternak et al. showed that MMP-8 and MMP-9 levels, as biomarkers of ischemia measured 4 h after surgery, were significantly increased in patients with AL [35]. However, a subsequent study did not find any statistically significant changes in MMP-9 levels in patients with AL on POD1, POD3, POD5 and POD7 [36].

Fouda et al. reported that intraperitoneal bacterial colonization might be an additional diagnostic tool that aids in the early detection of AL after CRS. They identified *Escherichia coli*, *Klebsiella* and *Pseudomonas species* in the drainage fluid of patients with AL on POD1; however, at least 24–48 h is required for detecting aerobic bacteria and 48–120 h for detecting anaerobic bacteria [9]. Semi-quantitative real-time polymerase chain reaction (RT-PCR) for *Enterococcus faecalis* in the drainage fluid was also demonstrated to be an objective, affordable, and fast screening tool for AL after CRS. However, a significant elevation in quantitative RT-PCR results was observed on POD2 [12].

Cytokines related to inflammation, such as TNF-α, interleukin (IL)−6, and IL-10, have also been evaluated in the drainage fluid of patients with AL. These cytokine levels were reported to increase significantly from POD1 to POD4 [34]. In this study, TNF-α levels significantly increased even on POD0 in the AL group and showed relatively good predictive performance for AL, although the predictive ability was not as strong as that of the ET assays. A significant increase in TNF-α levels in the drainage fluid in the AL group was not observed in the very early phase of surgery, especially on POD1 [9, 13, 37]. Most patients in previous studies underwent open surgery or had unknown details of the surgical procedure. Surgical stress itself influences peritoneal and plasma cytokine levels after CRS. Given that 93% of the patients (150/162) in our study underwent laparoscopic or robotic surgeries, a reduced inflammatory response observed in laparoscopic surgery [38] may have affected the TNF-α levels in the drainage fluid of the non-AL group. Thus, further studies are needed to determine whether other cytokines exhibit similarly good diagnostic performance.

In this study, the serum CRP levels on POD1 after CRS were significantly elevated in patients with AL. Previous meta-analyses have shown that PCT and CRP exhibit good negative predictive values for identifying patients at low risk for AL development after CRS on POD3 and POD5 [39]. The relatively low SOFA scores in patients with AL may have influenced our findings regarding the CRP and PCT levels.

The present study was associated with several limitations. First, the number of false-positive cases (*n* = 18) exceeding the ET-ESP cut-off value on POD0 was relatively high. A certain extent of contamination from the digestive tract into the peritoneal cavity during surgical procedures for anastomosis may have led to occasional elevation in ET levels on POD0 even in patients without AL. This occasional elevation of ET decreased time-dependently on POD1 and POD3 in patients without AL, which may account for the low sensitivity and specificity of ET-ESP and ET-TUB on POD0. This may have been due to inadequate surgical procedures and insufficient removal of ET contamination during intraperitoneal lavage at the end of the surgery. The procedural quality during surgery should be reviewed in future studies. Second, occasional contamination of the specimen may occur during sampling from the drainage bottle. Despite the expectation that the ET levels would gradually decrease in the non-AL group, an increase in the ET-ESP levels was observed on POD1 relative to POD0 in 20 of 154 patients with non-AL. Further elevation of the ET-ESP levels was also observed on POD3 in 21 of 154 patients with non-AL. These elevations in the ET-ESP levels caused by contamination in patients with non-AL were significantly lower than those on POD0 in patients with AL (data not shown). A false-positive error may be attributed to occasional sampling contamination on POD1 and POD3 in this study; however, the possibility of contamination in the specimen collected on POD0 (immediately postoperatively) is low. Therefore, it is reasonable to evaluate the ET-ESP levels in the drainage fluid on POD0 in future studies. Third, ET was not detected in the drainage fluid of patients without drainage or with poor drainage. Drain placement after CRS was performed at the surgeon’s discretion during the study period. One of the 14 patients without drain placement underwent sigmoidectomy, developed AL, and emergency relaparotomy on POD12 during the study period. There are differing opinions on drain placement after CRS. Several studies have questioned its use, noting no significant difference in the incidence of AL between patients with and without drains. In addition, the possibility of increased surgical site infections related to drain placement has also been suggested in these review articles [40–42]. However, according to our findings, drain placement may be worth considering for the prediction of AL on the first postoperative day, followed by early drain removal based on the defined criteria. Fourth, we only evaluated a small number of patients with non-identical diseases from a single center. Among the 38 patients who were unable to provide informed consent owing to dementia or other reasons, no patients developed AL. Although the number of patients with AL was lower than anticipated, we were still able to determine sufficient AUROC values for the ET-ESP and ET-TUB levels on POD0. Patients with CRC or IBD were included in this study; the incidence of AL was 3.7% in patients with CRC and 18.8% in those with IBD. These rates are consistent with previous reports [1, 4, 6–8, 43, 44]. Future multicenter, large-scale studies should focus on evaluating ET levels in drainage fluid of patients who underwent CRS for similar diseases, such as rectal cancer.

In conclusion, measurement of ET in drainage fluid using the ESP assay and an ET-specific LAL reagent can predict the development of AL in the early perioperative phase after CRS.

## References

[1] Chiarello MM, Fransvea P, Cariati M, Adams NJ, Bianchi V, Brisinda G. Anastomotic leakage in colorectal cancer surgery. Surg Oncol. 2022;40: 101708. 35092916 10.1016/j.suronc.2022.101708

[2] Nassar A, Challine A, O’Connell L, Voron T, Chafai N, Debove C, et al. Effective initial management of anastomotic leak in the maintenance of functional colorectal or coloanal anastomosis. Surg Today. 2023;53:718–27. 36385312 10.1007/s00595-022-02603-7

[3] Ha GW, Kim JH, Lee MR. Oncologic impact of anastomotic leakage following colorectal cancer surgery: a systematic review and meta-analysis (in eng). Ann Surg Oncol. 2017;24:3289–99. 28608118 10.1245/s10434-017-5881-8

[4] Lian L, Kiran RP, Remzi FH, Lavery IC, Fazio VW. Outcomes for patients developing anastomotic leak after ileal pouch-anal anastomosis: does a handsewn vs. stapled anastomosis matter? Dis Colon Rectum. 2009;52:387–93. 19333036 10.1007/DCR.0b013e31819ad4f2

[5] Luo WY, Singh S, Cuomo R, Eisenstein S. Modified two-stage restorative proctocolectomy with ileal pouch-anal anastomosis for ulcerative colitis: a systematic review and meta-analysis of observational research. Int J Colorectal Dis. 2020;35:1817–30. 32715346 10.1007/s00384-020-03696-7 PMC7733241

[6] Masuda T, Takamori H, Ogawa K, Shimizu K, Karashima R, Nitta H, et al. C-reactive protein level on postoperative day 3 as a predictor of anastomotic leakage after elective right-sided colectomy. Surg Today. 2022;52:337–43. 34370104 10.1007/s00595-021-02351-0

[7] Sahami S, Bartels SA, D’Hoore A, Fadok TY, Tanis PJ, Lindeboom R, et al. A multicentre evaluation of risk factors for anastomotic leakage after restorative proctocolectomy with ileal pouch-anal anastomosis for inflammatory bowel disease. J Crohns Colitis. 2016;10:773–8. 26417046 10.1093/ecco-jcc/jjv170

[8] Suzuki N, Yoshida S, Tomochika S, Nakagami Y, Shindo Y, Tokumitsu Y, et al. Determining the protective characteristics and risk factors for the development of anastomotic leakage after low anterior resection for rectal cancer. Surg Today. 2021;51:713–20. 33006668 10.1007/s00595-020-02133-0 PMC8055621

[9] Fouda E, El Nakeeb A, Magdy A, Hammad EA, Othman G, Farid M. Early detection of anastomotic leakage after elective low anterior resection. J Gastrointest Surg. 2011;15:137–44. 20978948 10.1007/s11605-010-1364-y

[10] Hirst NA, Tiernan JP, Millner PA, Jayne DG. Systematic review of methods to predict and detect anastomotic leakage in colorectal surgery. Colorectal Dis. 2014;16:95–109. 23992097 10.1111/codi.12411

[11] Hyman N, Manchester TL, Osler T, Burns B, Cataldo PA. Anastomotic leaks after intestinal anastomosis: it’s later than you think. Ann Surg. 2007;245:254–8. 17245179 10.1097/01.sla.0000225083.27182.85 PMC1876987

[12] Komen N, Slieker J, Willemsen P, Mannaerts G, Pattyn P, Karsten T, et al. Polymerase chain reaction for Enterococcus faecalis in drain fluid: the first screening test for symptomatic colorectal anastomotic leakage. The Appeal-study: analysis of parameters predictive for evident anastomotic leakage. Int J Colorectal Dis. 2014;29:15–21. 24122105 10.1007/s00384-013-1776-8

[13] Sammour T, Singh PP, Zargar-Shoshtari K, Su’a B, Hill AG. Peritoneal cytokine levels can predict anastomotic leak on the first postoperative day. Dis Colon Rectum. 2016;59:551–6. 27145313 10.1097/DCR.0000000000000598

[14] Shimizu T, Obata T, Sonoda H, Akabori H, Miyake T, Yamamoto H, et al. Diagnostic potential of endotoxin scattering photometry for sepsis and septic shock. Shock. 2013;40:504–11. 24089007 10.1097/SHK.0000000000000056

[15] Junger W, Junger WG, Miller K, Bahrami S, Redl H, Schlag G, et al. Early detection of anastomotic leaks after colorectal surgery by measuring endotoxin in the drainage fluid. Hepatogastroenterology. 1996;43:1523–9. 8975959

[16] Kambayashi J, Yokota M, Sakon M, Shiba E, Kawasaki T, Mori T, et al. A novel endotoxin-specific assay by turbidimetry with Limulus amoebocyte lysate containing beta-glucan. J Biochem Biophys Methods. 1991;22:93–100. 2061565 10.1016/0165-022x(91)90022-o

[17] Obata T, Nomura M, Kase Y, Sasaki H, Shirasawa Y. Early detection of the Limulus amebocyte lysate reaction evoked by endotoxins. Anal Biochem. 2008;373:281–6. 17980693 10.1016/j.ab.2007.09.018

[18] Shimizu T, Obata T, Sonoda H, Akabori H, Tabata T, Eguchi Y, et al. The ability of endotoxin adsorption during a longer duration of direct hemoperfusion with a polymyxin B-immobilized fiber column in patients with septic shock. Transfus Apher Sci. 2013;49:499–503. 23683501 10.1016/j.transci.2013.04.042

[19] Hashiguchi Y, Muro K, Saito Y, Ito Y, Ajioka Y, Hamaguchi T, et al. Japanese society for cancer of the colon and rectum (JSCCR) guidelines 2019 for the treatment of colorectal cancer. Int J Clin Oncol. 2020;25:1–42. 31203527 10.1007/s10147-019-01485-z PMC6946738

[20] Nakase H, Uchino M, Shinzaki S, Matsuura M, Matsuoka K, Kobayashi T, et al. Evidence-based clinical practice guidelines for inflammatory bowel disease 2020. J Gastroenterol. 2021;56:489–526. 33885977 10.1007/s00535-021-01784-1 PMC8137635

[21] Rahbari NN, Weitz J, Hohenberger W, Heald RJ, Moran B, Ulrich A, et al. Definition and grading of anastomotic leakage following anterior resection of the rectum: a proposal by the International Study Group of Rectal Cancer. Surgery. 2010;147:339–51. 20004450 10.1016/j.surg.2009.10.012

[22] Tsuchiya M, Takaoka A, Tokioka N. Matsuura S [Development of an endotoxin-specific Limulus amebocyte lysate test blocking beta-glucan-mediated pathway by carboxymethylated curdlan and its application]. Nihon Saikingaku Zasshi. 1990;45:903–11. 2082026 10.3412/jsb.45.903

[23] Kanda Y. Investigation of the freely available easy-to-use software “EZR” for medical statistics. Bone Marrow Transplant. 2013;48:452–8. 23208313 10.1038/bmt.2012.244 PMC3590441

[24] Faul F, Erdfelder E, Buchner A, Lang AG. Statistical power analyses using G*Power 3.1: tests for correlation and regression analyses. Behav Res Methods. 2009;41:1149–60. 19897823 10.3758/BRM.41.4.1149

[25] Kuhn F, Schardey J, Wirth U, Schiergens T, Crispin A, Beger N, et al. Endoscopic vacuum therapy for the treatment of colorectal leaks—a systematic review and meta-analysis. Int J Colorectal Dis. 2022;37:283–92. 34817647 10.1007/s00384-021-04066-7 PMC8803669

[26] Sparreboom CL, Wu ZQ, Ji JF, Lange JF. Integrated approach to colorectal anastomotic leakage: communication, infection and healing disturbances. World J Gastroenterol. 2016;22:7226–35. 27621570 10.3748/wjg.v22.i32.7226 PMC4997633

[27] Wu Z, Vakalopoulos KA, Boersema GS, Kroese LF, Lam KH, van der Horst PH, et al. The prevention of colorectal anastomotic leakage with tissue adhesives in a contaminated environment is associated with the presence of anti-inflammatory macrophages. Int J Colorectal Dis. 2014;29:1507–16. 25255850 10.1007/s00384-014-2012-x

[28] Gan X, Wong B, Wright SD, Cai TQ. Production of matrix metalloproteinase-9 in CaCO-2 cells in response to inflammatory stimuli. J Interferon Cytokine Res. 2001;21:93–8. 11244573 10.1089/107999001750069953

[29] Habara T, Nakatsuka M, Konishi H, Asagiri K, Noguchi S, Kudo T. The biological effects of antiadhesion agents on activated RAW264.7 macrophages. J Biomed Mater Res. 2002;61:628–33. 12115453 10.1002/jbm.10247

[30] Masure S, Proost P, Van Damme J, Opdenakker G. Purification and identification of 91-kDa neutrophil gelatinase. Release by the activating peptide interleukin-8. Eur J Biochem. 1991;198:391–8. 1645657 10.1111/j.1432-1033.1991.tb16027.x

[31] Ahrendt GM, Tantry US, Barbul A. Intra-abdominal sepsis impairs colonic reparative collagen synthesis. Am J Surg. 1996;171:102–7. 8554122 10.1016/S0002-9610(99)80082-8

[32] Edomskis P, Goudberg MR, Sparreboom CL, Menon AG, Wolthuis AM, D’Hoore A, et al. Matrix metalloproteinase-9 in relation to patients with complications after colorectal surgery: a systematic review. Int J Colorectal Dis. 2021;36:1–10. 32865714 10.1007/s00384-020-03724-6 PMC7782374

[33] Kimura Y, Nakao A, Tamura H, Tanaka S, Takagi H. Clinical and experimental studies of the limulus test after digestive surgery. Surg Today. 1995;25:790–4. 8555696 10.1007/BF00311454

[34] Su’a BU, Mikaere HL, Rahiri JL, Bissett IB, Hill AG. Systematic review of the role of biomarkers in diagnosing anastomotic leakage following colorectal surgery. Br J Surg. 2017;104:503–12. 28295255 10.1002/bjs.10487

[35] Pasternak B, Matthiessen P, Jansson K, Andersson M, Aspenberg P. Elevated intraperitoneal matrix metalloproteinases-8 and -9 in patients who develop anastomotic leakage after rectal cancer surgery: a pilot study. Colorectal Dis. 2010;12:e93–8. 19508511 10.1111/j.1463-1318.2009.01908.x

[36] Kostic Z, Panisic M, Milev B, Mijuskovic Z, Slavkovic D, Ignjatovic M. Diagnostic value of serial measurement of C-reactive protein in serum and matrix metalloproteinase-9 in drainage fluid in the detection of infectious complications and anastomotic leakage in patients with colorectal resection. Vojnosanit Pregl. 2015;72:889–98. 26665555 10.2298/vsp140723011k

[37] Yamamoto T, Umegae S, Matsumoto K, Saniabadi AR. Peritoneal cytokines as early markers of peritonitis following surgery for colorectal carcinoma: a prospective study. Cytokine. 2011;53:239–42. 21075004 10.1016/j.cyto.2010.10.006

[38] Kampman SL, Smalbroek BP, Dijksman LM, Smits AB. Postoperative inflammatory response in colorectal cancer surgery: a meta-analysis. Int J Colorectal Dis. 2023;38:233. 37725227 10.1007/s00384-023-04525-3

[39] Bona D, Danelli P, Sozzi A, Sanzi M, Cayre L, Lombardo F, et al. C-reactive protein and procalcitonin levels to predict anastomotic leak after colorectal surgery: systematic review and meta-analysis. J Gastrointest Surg. 2023;27:166–79. 36175720 10.1007/s11605-022-05473-z

[40] Denost Q, Rouanet P, Faucheron JL, Panis Y, Meunier B, Cotte E, et al. To drain or not to drain infraperitoneal anastomosis after rectal excision for cancer: the GRECCAR 5 randomized trial. Ann Surg. 2017;265:474–80. 27631776 10.1097/SLA.0000000000001991

[41] Karliczek A, Jesus EC, Matos D, Castro AA, Atallah AN, Wiggers T. Drainage or nondrainage in elective colorectal anastomosis: a systematic review and meta-analysis. Colorectal Dis. 2006;8:259–65. 16630227 10.1111/j.1463-1318.2006.00999.x

[42] Urbach DR, Kennedy ED, Cohen MM. Colon and rectal anastomoses do not require routine drainage: a systematic review and meta-analysis. Ann Surg. 1999;229:174–80. 10024097 10.1097/00000658-199902000-00003 PMC1191628

[43] Sagap I, Remzi FH, Hammel JP, Fazio VW. Factors associated with failure in managing pelvic sepsis after ileal pouch-anal anastomosis (IPAA)–a multivariate analysis. Surgery. 2006;140:691–703. 17011918 10.1016/j.surg.2006.07.015

[44] Wada H, Tominaga T, Nonaka T, To K, Hamada K, Araki M, et al. Charlson comorbidity index predicts anastomotic leakage in patients with resected right-sided colon cancer. Surg Today. 2022;52:804–11. 35165757 10.1007/s00595-022-02472-0

